# Improvement of Ylang-Ylang Essential Oil Characterization by GC×GC-TOFMS

**DOI:** 10.3390/molecules18021783

**Published:** 2013-01-30

**Authors:** Michał Brokl, Marie-Laure Fauconnier, Céline Benini, Georges Lognay, Patrick du Jardin, Jean-François Focant

**Affiliations:** 1 Chemistry Department—CART, Organic and Biological Analytical Chemistry, University of Liège, Allée du 6 Août B6c, Liège B-4000, Belgium; E-Mail: M.Brokl@ulg.ac.be; 2 Plant Biology Unit, Gembloux Agro-Bio Tech, University of Liège, Passage des Déportés 2, Gembloux B-5030, Belgium; E-Mails: Marie-Laure.Fauconnier@ulg.ac.be (M.-L.F.); Celine.Benini@gmail.com (C.B.); Patrick.Dujardin@ulg.ac.be (P.J.); 3 Analytical Chemistry Laboratory-CART, Gembloux Agro-Bio Tech, University of Liège, Passage des des Déportés 2, Gembloux B-5030, Belgium; E-Mail: Georges.Lognay@ulg.ac.be

**Keywords:** ylang-ylang, *Cananga odorata*, essential oil, comprehensive two-dimensional gas chromatography (GC×GC), time-of-flight mass spectrometry (TOFMS)

## Abstract

A single fraction of essential oil can often contain hundreds of compounds. Despite of the technical improvements and the enhanced selectivity currently offered by the state-of-the-art gas chromatography (GC) and mass spectrometry (MS) instruments, the complexity of essential oils is frequently underestimated. Comprehensive two-dimensional GC coupled to time-of-flight MS (GC×GC-TOFMS) was used to improve the chemical characterization of ylang-ylang essential oil fractions recently reported in a previous one-dimensional (1D) GC study. Based on both, the enhanced chromatographic separation and the mass spectral deconvolution, 161 individual compounds were identified and labeled as potentially characteristic analytes found in both low and high boiling fractions issued from distillation of mature ylang-ylang flowers. Compared to the most recent full GC-MS characterization, this represents 75 new compounds, essentially consisting of terpenes, terpenoid esters, and alcohols.

## 1. Introduction

Ylang-ylang essential oil, together with jasmine, rose and neroli, is one of the few essential oils extracted from flowers that are exploited at a large scale. Ylang-ylang essential oil is distilled from the mature fresh flowers of the *Annonaceae* family tropical tree *Cananga odorata* [Lam.] Hook f. and Thomson forma *genuina*. The plant originates from the Indonesian archipelago, but is currently exploited in the Western Indian Ocean islands, mainly in Comoros Islands, Mayotte and Madagascar [1,2]. Ylang-ylang essential oil is mainly used by the cosmetic industry in applications ranging from high grade perfume conception to soap manufacture, but also, to a lesser extent, in aromatherapy or even as a food ingredient [1,3,4,5]. In addition to a great cultural and tourism value of the plant, the production of ylang-ylang essential oil plays an important economic role as the oil represents the second most important export product for the Comoros Islands, after clovers [1,6].

Ylang-ylang essential oil production has the particularity of relying on a fractionation based on distillation times, resulting in four to five grades of oil that have different commercial applications. Commercial grades strongly differ in their chemical composition, the first fraction being richer in very volatile compounds like esters, aldehydes or alcohols, while the last fraction is richer in less volatile compounds like sesquiterpenes [2]. In the past, the chemical composition of ylang-ylang essential oil fractions has been studied by gas chromatography coupled to mass spectrometry (GC-MS), especially quadrupole analyzers, revealing its complexity [4,7]. This permitted researchers to highlight the major components of the oil and use them for quality and/or origin control. In the aroma and flavor industries, the standard published by the French standardization system [AFNOR, ISO 3063:2004(E)] is considered to be the reference. This ISO standard is based on the measurement and comparison of 15 major compounds, but only allows distinguishing between two groups of essential oils, based on geographic origin: Mayotte/Comoros Islands and Madagascar [8].

Despite its economic and social importance, little is known about the chemical variability of ylang-ylang essential oil and factors potentially causing it. In a previous work based on the monitoring of the 15 “AFNOR” compounds, we highlighted significant variations in oil compositions between islands, but also within plantations on a same island. Moreover, the genetic differentiation pattern was shown to be different from the chemical differentiation pattern, which indicated a possible important environmental effect (climate, edaphic conditions, soil composition, solar exposition and agronomic practices) [9]. To refine those findings, we recently carried out a deeper and more exhaustive GC-MS investigation of the chemical characterization of four fractions of ylang-ylang essential oil distillated in controlled conditions in four different locations (Grand Comore, Mayotte, Nosi Bé and Ambanja). As a result, a total of 119 potentially characteristic compounds were reported, among which 32 compounds had never been reported before. Quantification performed by GC coupled to flame ionization detection (FID) further allowed us to build regression trees that permitted to differentiate the four geographic origins for the four fractions [2]. The chemical polymorphism that was highlighted in that study represents an interesting tool for perfumers and flavorists in search of new specific raw material for their compositions.

Comprehensive two-dimensional gas chromatography (GC×GC) is an already well-established technique used to perform separations of highly complex mixtures of GC-amenable compounds [10,11,12]. It has successfully been applied for the analysis of specific essential oils [13,14,15]. As compared to one-dimensional gas chromatography (1DGC), the use of two separation mechanisms results in a significant increase of peak capacity, whereas modulation can provide an improvement in sensitivity [16]. Coupling this technique to time-of-flight mass spectrometer (TOFMS), capable of acquiring up to 500 full-range spectra per second [17], offers the possibility of mass deconvolution—an additional tool to resolve coelutions in the mass spectral domain.

Based on the complexity of the 1DGC chromatogram generated in our previous study [2], we investigated the use of GC×GC-TOFMS for a more exhaustive analysis of selected ylang-ylang essential oil fractions with the aim of improving the differentiation approach. For this conceptual study, we focused our efforts on the first and the last fractions issued from the distillation of an ylang-ylang essential oil produced from fresh flowers collected in Mayotte.

## 2. Results and Discussion

**Figure 1:**
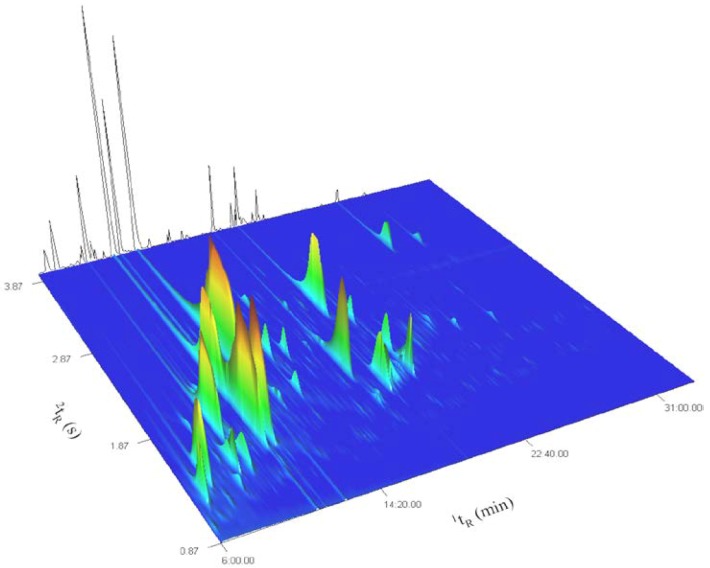
GC×GC TOFMS TIC surface plot of fraction 1 of ylang-ylang essential oil.

Figure 1 represents the total ion current (TIC) surface plot of the signal recorded for the most volatile fraction of the ylang-ylang essential oil from Mayotte (fraction 1). The background contour line represents the reconstructed 1D trace, issued from the summation of chromatographic signals collected along the second dimension retention time axis (^2^t_R_), and reflects the separation that could be achieved using classical 1DGC.

Several 1DGC coelutions are visible and the number of peaks separated in the two-dimensional chromatographic space clearly surpasses the number of peaks separated in the reconstructed 1DGC trace. This includes situations where peaks are present at levels that differ of several orders of magnitude, for which the identification of the low level coeluters would have been particularly difficult, even with the help of mass spectral deconvolution. The use of the combination of a low polar 5% phenyl phase for the first dimension (^1^D) with a medium polar 50% phenyl polysilphenylene phase for the second dimension (^2^D) allowed an efficient use of the available chromatographic space. Peak widths at half height in ^2^D ranged from 100 ms to 150 ms and were distributed over 3 s of the 4 s modulation period (P_M_). Tailing was observed for some of the most abundant compounds in ^2^D. This resulted in a slight wrap-around of the tails but did not practically affect the separation efficiency as it did not create coelution issues.

**Figure 2:**
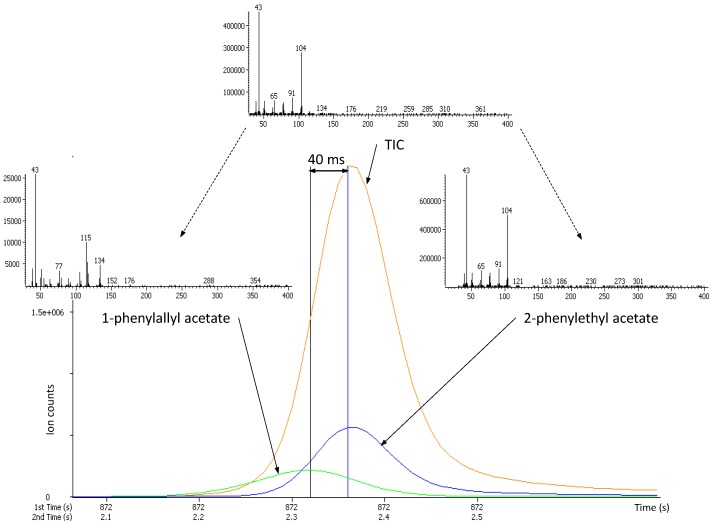
Deconvoluted ion current (DIC) traces for two coeluting esters. DICs were reconstructed based on unique masses of *m/z* 115 for 1-phenylallyl acetate and *m/z* 104 for 2-phenylethyl acetate; the signal for 1-phenylallyl acetate has been magnified 20 times for clarity. The mass spectrum on the top represents the raw data. The mass spectra on the left and on the right represent the deconvoluted signals of 1-phenylallyl acetate and 2-phenylethyl acetate, respectively.

Several hundred peaks were detected and their deconvoluted mass spectral signatures were processed against mass spectrometry libraries. In several cases, it appeared that, despite the use of two chromatographic separation axes, some peaks were still coeluting and were separated by mass spectral deconvolution. The TOFMS acquisition rate of 100 spectra s^−1^ permitted to differentiate between peaks with the same first dimension retention times (^1^t_R_) (identical linear retention indices (*I*^T^)) but exhibiting slight differences in ^2^t_R_ values. Figure 2 illustrates such a case (^1^t_R_(A) = ^1^t_R_(B), ^2^t_R_(A) = 2.32 s and ^2^t_R_(B) = 2.36 s) where the peak apexes of compounds A and B were only 40 ms apart of each other.

In such a critical scenario (only four full mass spectra acquired in between peak apexes), the deconvolution software was able to separate two MS signals and further successively identified them by mass library searching (forward-reverse similarities of 826–897 and forward-reverse similarities of 935–952, respectively for compound A and B). The lower score of library matching for compound A (1-phenylallyl acetate), compared to compound B (2-phenylethyl acetate) is, most probably, to be related to the much lower concentration, resulting in lowering the intensity of the MS signal.

After first data processing, peak tables for fractions 1 and 4 accounted for 681 and 839 hits, respectively. During the second processing, artifacts and column bleeding were removed, leaving 370 and 446 hits for fractions 1 and 4, respectively. From those, 161 individual compounds were identified and labeled as potentially characteristic analytes found in either fraction 1 or 4. This selection was based on manual review of the large peak tables, focusing on signals that exhibited signal to noise ratio (S/N) values greater than 200, and forward and reverse library match similarity factor over 750 and 800, respectively. Selected compounds, along with their calculated *I*^T^, ^1^t_R_, ^2^t_R_, molecular formula, and a relative abundance are listed in Table 1. As a comparison, the previous GC-MS study [2] reported 96 compounds present in either fraction 1 or 4. Amongst those 96 compounds, 79 were also found in the list of 161 compounds generated from GC×GC-TOFMS analysis.

**Table 1:** Selected compounds from fractions 1 and 4 of ylang-ylang essential oil.

**No.**	**Name**	***I*****^T^**	**^1^tR (min:s), ^2^tR (s)**	**Formula**	**Relative abundance %**	
					Fraction 1	Fraction 4
**1**	3-hexen-1-ol ^#^	867	6:16, 1.28	C_6_H_12_O	tr	-
**2**	3-methyl-3-buten-1-ol acetate	885	6:36, 1.26	C_7_H_12_O_2_	2.16	0.22
**3**	heptanal	907	7:00, 1.29	C_7_H_14_O	0.05 ^†^	-
**4**	3-methyl-2-butenyl acetate	923	7:16, 1.38	C_7_H_12_O_2_	4.24	0.32
**5**	*α*-pinene	941	7:36, 1.17	C_10_H_16_	0.1	0.04
**6**	benzaldehyde	967	8:04, 1.87	C_7_H_6_O	0.21	0.05
**7**	sabinene ^#^	978	8:16, 1.27	C_10_H_16_	tr	-
**8**	6-methyl-5-hepten-2-one	986	8:24, 1.52	C_8_H_14_O	0.07	0.03 ^†^
**9**	*β*-myrcene	993	8:32, 1.27	C_10_H_16_	0.29	tr
**10**	decane *	1000	8:40, 1.13	C_10_H_22_	tr	0.03
**11**	( *3Z*)-3-hexenyl acetate	1003	8:44, 1.47	C_8_H_14_O_2_	0.5	0.03
**12**	*n*-hexyl acetate	1009	8:52, 1.42	C_8_H_16_O_2_	0.97	0.07
**13**	*α*-phellandrene *	1012	8:56, 1.33	C_10_H_16_	tr	tr
**14**	*p*-cresyl methyl ether	1025	9:12, 1.88	C_8_H_10_O	9.7	1.63
**15**	*β*-limonene*	1034	9:24, 1.36	C_10_H_16_	0.13	0.15
**16**	1,8-cineole	1037	9:28, 1.45	C_10_H_18_O	1.11	0.22
**17**	benzyl alcohol	1037	9:28, 2.13	C_7_H_8_O	0.53	tr
**18**	*β*-ocimene *	1046	9:40, 1.38	C_10_H_16_	0.07	tr
**19**	ester (MW 174)	1046	9:40, 1.71	C_8_H_14_O_4_	0.28	tr
**20**	phenyl acetaldehyde	1046	9:40, 2.16	C_8_H_8_O	0.17	tr ^†^
**21**	*p*-cresol	1070	10:12, 2.10	C_7_H_8_O	0.19	0.05
**22**	*cis*-linalool oxide (furanoid)	1073	10:16, 1.53	C_10_H_18_O_2_	0.02	-
**23**	*trans*-linalool oxide (furanoid)	1088	10:36, 1.59	C_10_H_18_O_2_	tr ^†^	-
**24**	2-methoxyphenol	1089	10:36, 2.18	C_7_H_8_O_2_	0.7	tr
**25**	terpinolene ^#^	1097	10:48, 1.78	C_10_H_16_	0.07	-
**26**	methyl benzoate	1098	10:48, 2.25	C_8_H_8_O_2_	6.05	0.69
**27**	undecane *	1100	10:36, 1.24	C_11_H_24_	-	tr
**28**	linalool	1100	10:52, 1.76	C_10_H_18_O	8.95	0.34
**29**	levoglucosenone *	1101	10:52, 3.22	C_6_H_6_O_3_	tr	0.07
**30**	monoterpene (MW 136) *	1112	11:08, 1.34	C_10_H_16_	0.05	0.42
**31**	methyl 3-methylbutanoate *	1117	11:16, 1.58	C_6_H_12_O_2_	tr	-
**32**	methyl caprylate *	1123	11:24, 1.54	C_9_H_18_O_2_	0.43	7.24
**33**	*α*-pyronene *	1126	11:28, 1.52	C_10_H_16_	0.04	-
**34**	plinol A *	1132	11:36, 1.67	C_10_H_18_O	0.14	-
**35**	monoterpene (MW 136) *	1134	11:40, 1.39	C_10_H_16_	0.03	0.17
**36**	phenylacetonitrile	1138	11:44, 2.79	C_8_H_7_N	0.02	tr
**37**	veratrole	1141	11:48, 2.39	C_8_H_10_O_2_	0.07	tr ^†^
**38**	plinol D *	1149	12:00, 1.73	C_10_H_18_O	0.05	-
**39**	1-phenyl-2-propen-1-ol *	1152	12:04, 2.28	C_9_H_10_O	tr	tr
**40**	benzyl acetate	1167	12:24, 2.57	C_9_H_10_O_2_	27.48	0.07
**41**	ethyl benzoate	1175	12:36, 2.15	C_9_H_10_O_2_	0.42	-
**42**	2-methoxy-4-methylphenol	1192	13:00, 2.29	C_8_H_10_O_2_	0.03	tr
**43**	methyl salicylate	1198	13:08, 2.30	C_8_H_8_O_3_	0.32	0.15
**44**	dodecane *	1200	13:12, 1.27	C_12_H_26_	tr	tr
**45**	*α*-terpineol	1200	13:12, 1.88	C_10_H_18_O	0.26	tr
**46**	methyl chavicol	1201	13:12, 2.10	C_10_H_12_O	0.06	-
**47**	1-methoxy-4-propylbenzene *	1209	13:24, 1.96	C_10_H_14_O	tr	tr
**48**	nerol ^#^	1225	13:48, 1.82	C_10_H_18_O	tr	-
**49**	linalyl acetate ^#^	1249	14:24, 1.63	C_12_H_20_O_2_	0.03	-
**50**	geraniol	1249	14:24, 1.92	C_10_H_18_O	0.36	tr
**51**	1-phenylallyl acetate *	1255	14:32, 2.32	C_11_H_12_O_2_	tr	tr
**52**	2-phenylethyl acetate	1255	14:32, 2.36	C_10_H_12_O_2_	0.57	tr
**53**	4-methoxy benzaldehyde *	1261	14:40, 2.90	C_8_H_8_O_2_	tr	-
**54**	geranial	1268	14:52, 2.04	C_10_H_16_O	0.03	tr ^†^
**55**	diethyl 1,5-pentanedioate *	1274	15:00, 2.14	C_9_H_16_O_4_	tr	tr
**56**	*trans*-anethol	1290	15:24, 2.31	C_10_H_12_O	0.36	tr
**57**	1 *H*-indole *	1299	15:36, 3.28	C_8_H_7_N	tr	tr
**58**	2-phenylnitroethane	1301	15:40, 2.96	C_8_H_9_NO_2_	0.25	0.03
**59**	vinyl butyrate *	1309	15:52, 2.07	C_6_H_10_O_2_	tr	tr
**60**	cinnamyl alcohol	1309	15:52, 2.81	C_9_H_10_O	tr	tr ^†^
**61**	*p*-vinylguaiacol	1314	16:00, 2.56	C_9_H_10_O_2_	tr ^†^	tr
**62**	sesquiterpene (MW 204) *	1318	15:48, 2.11	C_15_H_24_	-	tr
**63**	diethyl ( *2E*)-3-methyl-2-pentanedioate *	1325	16:16, 2.17	C_10_H_16_O_4_	tr	0.02
**64**	2,5-dimethyl-3-methylene-1,5-heptadiene *	1330	16:24, 1.54	C_10_H_16_	tr	0.04
**65**	methyl 2-methoxybenzoate ^#^	1334	16:28, 2.97	C_9_H_10_O_3_	tr	tr
**66**	bicycloelemene	1338	16:36, 1.58	C_15_H_24_	0.03	0.29
**67**	5-indanol	1339	16:36, 2.66	C_9_H_10_O	tr	tr
**68**	ester (MW 190) *	1341	16:40, 2.14	C_12_H_14_O_2_	tr	tr
**69**	benzyl butyrate	1347	16:48, 2.32	C_11_H_14_O_2_	0.06	tr ^†^
**70**	methyl 2-aminobenzoate *	1347	16:48, 2.99	C_8_H_9_NO_2_	tr	tr
**71**	*α*-cubebene*	1354	17:00, 1.53	C_15_H_24_	tr	0.09
**72**	eugenol	1355	17:00, 2.47	C_10_H_12_O_2_	tr	-
**73**	benzenepropanol, acetate *	1371	17:24, 2.39	C_11_H_14_O_2_	0.03	tr
**74**	neryl acetate	1373	17:28, 1.95	C_12_H_20_O_2_	2.74	0.21
**75**	geranyl acetate	1376	17:32, 2.00	C_12_H_20_O_2_	2	-
**76**	methyl 4-methoxybenzoate	1377	17:32, 2.79	C_9_H_10_O_3_	0.08	tr
**77**	*α*-ylangene	1378	17:16, 1.67	C_15_H_24_	-	0.06
**78**	butyl benzoate	1376	17:32, 2.31	C_11_H_14_O_2_	0.04	tr ^†^
**79**	*α*-copaene	1384	17:44, 1.59	C_15_H_24_	0.11	0.76
**80**	sesquiterpene (MW 204) *	1389	17:32, 1.72	C_15_H_24_	-	0.07
**81**	*β*-bourbonene	1392	17:56, 1.64	C_15_H_24_	tr ^†^	tr
**82**	*β*-cubebene	1395	18:00, 1.64	C_15_H_24_	0.13	0.56
**83**	vanillin	1399	18:04, 3.30	C_8_H_8_O_3_	tr	0.05
**84**	tetradecane *	1400	18:08, 1.35	C_14_H_30_	tr	tr
**85**	sesquiterpene (MW 204) *	1403	17:52, 1.71	C_15_H_24_	-	tr
**86**	2-methoxy-4-(1-propenyl)-phenol *	1407	18:16, 2.59	C_10_H_12_O_2_	tr	tr
**87**	*p*-anisyl acetate	1418	18:32, 2.77	C_10_H_12_O_3_	0.04	tr
**88**	*β*-ylangene *	1429	18:48, 1.79	C_15_H_24_	1.71	0.73
**89**	*β*-copaene	1440	19:04, 1.78	C_15_H_24_	0.72	0.12
**90**	sesquiterpene (MW 204) *	1442	18:48, 1.88	C_15_H_24_	-	7.48
**91**	cinnamyl acetate	1447	19:12, 2.78	C_11_H_12_O_2_	0.9	1.59
**92**	3-methyl-3-butenyl benzoate *	1449	19:16, 2.37	C_12_H_14_O_2_	0.03	tr
**93**	isoeugenol	1452	19:20, 2.65	C_10_H_12_O_2_	0.63	0.38
**94**	*β*-caryophyllene	1455	19:24, 1.79	C_15_H_24_	0.37	0.3
**95**	aromandendrene*	1460	19:32, 1.79	C_15_H_24_	0.06	1.53
**96**	*α*-humulene	1467	19:24, 1.96	C_15_H_24_	-	6.2
**97**	isogermacrene-D	1472	19:48, 1.77	C_15_H_24_	0.03	1.83
**98**	*α*-ionene*	1483	20:04, 1.78	C_13_H_18_	tr ^†^	tr
**99**	germacrene-D	1495	20:20, 1.80	C_11_H_22_O	tr	2.76
**100**	3-methyl-2-butenyl benzoate	1492	20:16, 2.47	C_12_H_14_O_2_	0.39	0.21
**101**	pentadecane*	1500	20:28, 1.41	C_15_H_32_	tr	tr
**102**	( *Z,E*)-*α*-farnesene*	1503	20:16, 1.87	C_15_H_24_	-	0.19
**103**	*α*-muurolene	1503	20:16, 2.03	C_15_H_24_	-	0.31
**104**	( *E,E*)-*α*-farnesene	1506	20:36, 1.79	C_15_H_24_	1.62	10.1
**105**	*β*-curcumenene	1512	20:44, 1.90	C_15_H_24_	0.39	2.73
**106**	*γ*-cadinene	1521	20:56, 1.88	C_15_H_24_	tr	2.14
**107**	*δ*-cadinene	1527	21:04, 1.92	C_15_H_24_	0.28	0.61
**108**	guaiacyl acetone *	1528	21:04, 3.26	C_10_H_12_O_3_	tr	tr
**109**	zonarene	1547	21:32, 1.87	C_15_H_24_	tr ^†^	tr
**110**	sesquiterpene (MW 202) *	1540	21:04, 2.05	C_15_H_22_	-	tr
**111**	benzyl 4-methylpentanoate *	1548	21:32, 2.33	C_13_H_18_O_2_	tr	tr
**112**	elemol	1556	21:44, 1.99	C_15_H_26_O	tr	0.02
**113**	*cis*-3-hexenyl benzoate	1577	22:12, 2.37	C_13_H_16_O_2_	tr	tr
**114**	germacren D-4-ol *	1589	22:28, 2.02	C_15_H_26_O	0.06	tr
**115**	caryophyllene oxide	1595	22:36, 2.17	C_15_H_24_O	0.06	tr
**116**	hexadecane *	1600	22:24, 1.51	C_16_H_34_	tr	tr
**117**	guaiol	1607	22:52, 2.05	C_15_H_26_O	tr	0.46
**118**	isoeugenol acetate	1607	22:52, 2.89	C_12_H_14_O_3_	tr	tr
**119**	oxygenated sesquiterpene (MW 220) *	1625	22:56, 2.22	C_15_H_24_O	-	0.06
**120**	sesquiterpene (MW 206) *	1625	22:56, 2.70	C_15_H_26_	-	tr
**121**	copaborneol	1637	23:32, 2.11	C_15_H_26_O	tr	0.03
**122**	sesquiterpene (MW 202) *	1643	23:20, 2.33	C_15_H_22_	-	tr
**123**	*τ*-muurolol	1652	23:52, 2.17	C_15_H_26_O	0.06	4.43
**124**	*α*-cadinol	1664	24:08, 2.23	C_15_H_26_O	0.07	1.52
**125**	oxygenated sesquiterpene (MW 222) *	1671	23:56, 2.58	C_15_H_26_O	-	tr
**126**	bulnesol *	1673	24:00, 2.34	C_15_H_26_O	tr	0.05
**127**	sesquiterpene (MW 200) *	1674	24:00, 2.63	C_15_H_20_	-	tr
**128**	farnesene*	1679	24:08, 2.35	C_15_H_24_	-	0.11
**129**	sesquiterpene (MW 204) *	1682	24:12, 2.01	C_15_H_24_	-	0.02
**130**	( *2Z,6E*)-farnesol *	1686	24:16, 2.37	C_15_H_26_O	-	0.02
**131**	sesquiterpene (MW 204)*	1686	24:16, 2.76	C_15_H_24_	-	0.02
**132**	cetene*	1694	24:48, 1.52	C_16_H_32_	tr	0.04
**133**	oxygenated sesquiterpene (MW 222)*	1695	24:28, 2.49	C_15_H_26_O	-	tr
**134**	ester (MW 196)*	1704	24:40, 1.99	C_12_H_20_O_2_	-	tr
**135**	globulol*	1708	24:44, 2.53	C_15_H_26_O	-	tr
**136**	( *2Z,6Z*)-farnesol*	1717	25:16, 2.10	C_15_H_26_O	0.09	1.43
**137**	sesquiterpene (MW 206)*	1724	25:04, 2.57	C_15_H_26_	-	tr
**138**	ledane*	1732	25:36, 1.70	C_15_H_26_	tr	0.15
**139**	( *2E,6E*)-farnesal*	1739	25:44, 2.20	C_15_H_24_O	tr	tr
**140**	(2 *E,6E*)-farnesol	1741	25:24, 2.49	C_15_H_26_O	-	0.03
**141**	benzyl benzoate	1776	26:28, 3.22	C_14_H_12_O_2_	0.97	1.24
**142**	*cis*-2-methyl-7-octadecene*	1794	26:52, 1.54	C_19_H_38_	tr	0.05
**143**	octadecane*	1800	27:00, 1.49	C_18_H_38_	tr	tr
**144**	octadecanal*	1818	27:20, 1.83	C_18_H_36_O	tr	0.02
**145**	( *2E,6E*)-farnesyl acetate	1832	27:36, 2.07	C_17_H_28_O_2_	0.05	2.05
**146**	*cis*-*Z*-*α*-bisabolene epoxide*	1875	28:04, 2.95	C_15_H_24_O	-	tr
**147**	benzyl salicylate	1881	28:32, 3.18	C_14_H_12_O_3_	0.21	4.18
**148**	nonadecane *	1900	28:56, 1.51	C_19_H_40_	tr	tr
**149**	hexadecanoic acid *	1958	29:40, 2.10	C_16_H_32_O_2_	-	0.27
**150**	geranyl benzoate	1965	29:48, 2.72	C_17_H_22_O_2_	-	tr
**151**	eicosane ^#^	2000	30:48, 1.54	C_20_H_42_	tr	tr
**152**	heneicosane *	2100	32:36, 1.57	C_21_H_44_	tr	tr
**153**	benzyl cinnamate	2102	32:16, 3.80	C_16_H_14_O_2_	-	tr
**154**	docosane *	2200	34:20, 1.59	C_22_H_46_	tr	tr
**155**	tricosane	2300	35:56, 1.62	C_23_H_48_	tr	tr
**156**	tetracosane	2400	37:32, 1.66	C_24_H_50_	tr	tr
**157**	pentacosane *	2500	39:04, 1.70	C_25_H_52_	tr	tr
**158**	hexacosane *	2600	40:32, 1.74	C_26_H_54_	tr	tr
**159**	heptacosane *	2700	41:56, 1.79	C_27_H_56_	tr	tr
**160**	octacosane *	2800	43:16, 1.86	C_28_H_58_	tr	-
**161**	nonacosane *	2900	44:36, 2.02	C_29_H_60_	tr	-

^#^ compound not detected by 1DGC analysis in neither of fractions 1 nor 4, but found by GC×GC analysis; * compound not detected before in ylang-ylang essential oil; ^†^ compound not detected by 1DGC in a specified fraction but found by GC×GC analysis; tr (trace): relative content < 0.02%; all identifications are based on retention indices, mass spectra and zone of elution.

A first observation is that 82 extra potentially characteristic analytes were thus reported. It is interesting to note that, among this group of 82 analytes, seven were already reported in either fraction 2 or 3 in the classical GC-MS study [2]. Such crossed presence of analytes in various fractions could potentially challenge the discrimination between fractions. So it is for the fact that, as 79 identical compounds were found in either fraction 1 or 4 from both GC and GC×GC studies, 17 compounds reported in the GC-MS study were not identified in the GC×GC study. Among these 17 compounds reported in the GC-MS study, three were confirmed by standard injections (3-methyl-2-buten-1-ol, 3-methyl butyl acetate, and *p*-cresyl acetate). None of the identities of the other missing compounds had been strictly verified, so that they could have been identified as other analytes in the GC×GC study. Moreover, 50% of them were reported at trace level (relative abundance below 0.02%), making proper identification quite difficult. Other factors such as chromatographic coelutions, the lack of deconvolution, the presence of interferences in MS data, and the use of slightly different mass spectral libraries could also be involved in this difference.

From the 75 (82−7) compounds that were not reported in the earlier study, the majority belonged to terpenes, terpenoid esters or alcohols, while 14 of them were alkanes that are not responsible for the aroma of essential oil. Some of the analytes e.g., 3-hexen-1-ol, sabinene or terpinolene were not found by 1DGC in neither of fractions 1 nor 4, but were detected in the present study; others like *β*-bourborene, *p*-vinylguaiacol or zonarene were missing in fraction 1, while 6-methyl-2-buten-1-yl acetate, phenylacetaldehyde or geranial, among others, were absent in fraction 4, but identified in the GC×GC analysis (Table 1).

**Figure 3:**
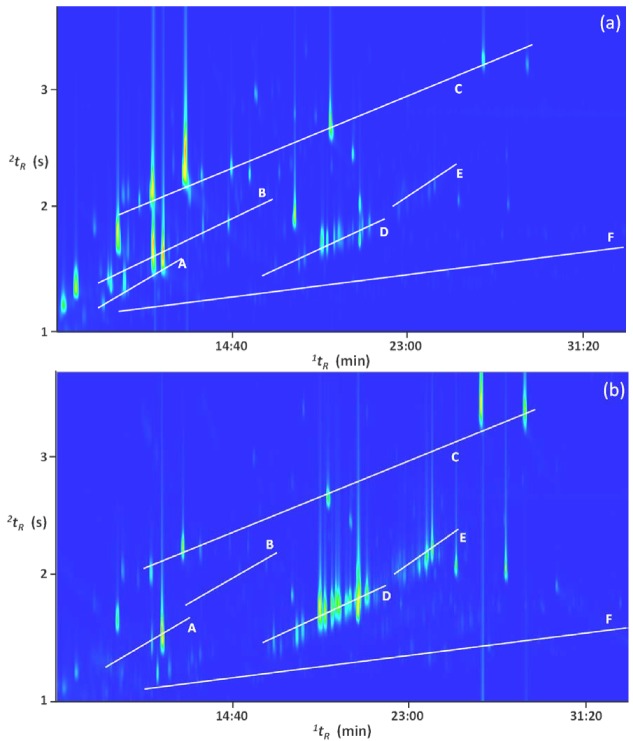
GC×GC total ion chromatogram (TIC) contour plots of two distillate fractions (**a**) collected after 25 min (fraction 1) and (**b**) after 8 h (fraction 4) of distillation of mature ylang-ylang flowers harvested in Mayotte. Letters A, B, C, D, E, and F correspond to monoterpenes, oxygenated monoterpenes, esters, sesquiterpenes, oxygenated sesquiterpenes, and alkanes, respectively.

Figure 3 shows the differences between the two distillate fractions collected after 25 min (a): fraction 1 and 8 h (b): fraction 4 of distillation of mature ylang-ylang flowers harvested in Mayotte. As already mentioned, fraction 4 is richer in high boiling compounds while fraction 1 is richer in more volatile ones.

**Figure 4:**
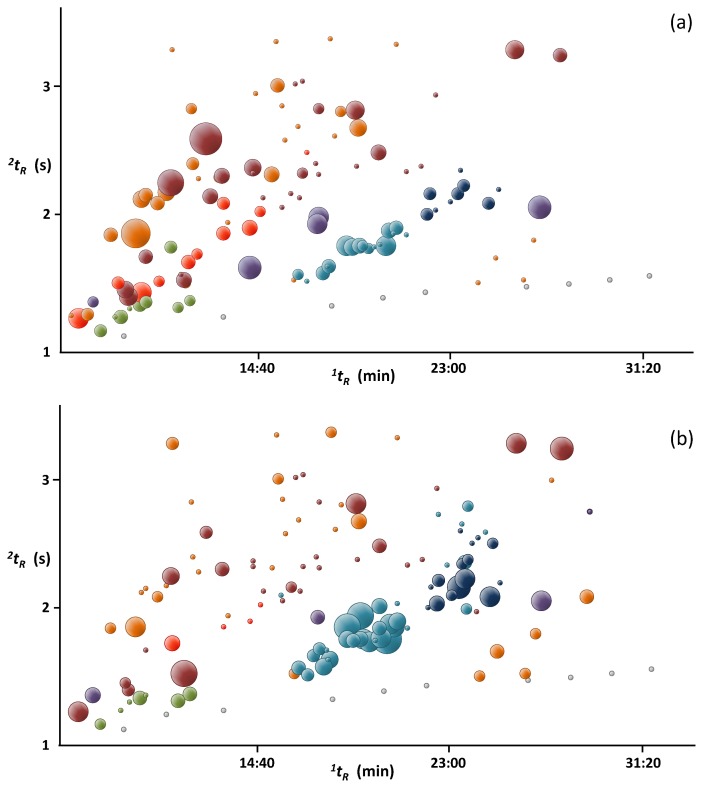
GC×GC bubble plots of two distillate fractions (**a**) collected after 25 min (fraction 1) and (**b**) after 8 h (fraction 4) of distillation of mature ylang-ylang flowers harvested in Mayotte. Chemical classes: alkanes—grey; monoterpenes—green; oxygenated monoterpenes—red; terpenoid esters—purple; sesquiterpenes—light blue; oxygenated sesquiterpenes—dark blue; esters—dark red; others—orange. The size of the bubbles reflects the relative abundances found in Table 1. Alkanes C_22_–C_29_are not shown.

Bubble plots presented in Figure 4 further illustrate the differences between the two fractions in terms of relative abundances of the major families of compounds present in the oil. GC×GC analysis allowed much better separation and led to easier identification as compounds with similar chemical properties eluted forming groups in the retention space. Several sesquiterpenes, present especially in fraction 4, could not be identified due to lack of standards and their mass spectra similarity.

Thus, further investigation is necessary to fully characterize the ylang-ylang essential oil. One field of efforts is the writing of specific scripts [18,19,20] that could allow reprocessing of data to highlight chromatographic zones specific to certain families of compounds and enhance the detectability of low abundance analytes.

## 3. Experimental

### 3.1. Plant Material and Essential Oil Distillation

Fresh, mature flowers of *C. odorata* were collected between 7 am and 8 am in July 2009 in Mavigoni, Mayotte. Seven trees were randomly selected in the plantation and the flowers were pooled to make one composite sample for the distillation, which was performed directly on the field with portable equipment, within an hour of the harvest (350 g of mature flowers from seven trees; 50 g of flowers collected from each tree). The flowers were subjected to hydrodistillation for 8 h using a Clevenger-type apparatus (equipped with a 2 L reactor and a 235 mm vertical column). The flowers were added to the water at 70 °C and then brought to the boil. Four separate fractions of the distillate were collected after 25 min (fraction 1), 1 h (fraction 2), 3 h (fraction 3), and 8 h (fraction 4). The essential oils were dried over anhydrous sodium sulfate (0.20 g of anhydrous sodium sulfate for 1.5 mL of essential oil) and kept in amber vials at 4 °C until use. The essential oils were diluted in diethyl ether (4 µL essential oil in 174 µL diethyl ether) containing 2 µL methyl octanoate standard (Sigma-Aldrich, St. Louis, MO, USA) previously diluted in diethyl ether (25 µL standard in 75 µL diethyl ether) Further details on solvents and consumables are available in a previous report [2]. The present study was carried out on fraction 1 and fraction 4.

### 3.2. GC×GC-TOFMS Parameters

The GC×GC TOFMS system consisted of an Agilent 7890 (Agilent Technologies, Palo Alto, CA, USA) gas chromatograph and a Pegasus 4D TOFMS (LECO, St. Joseph, MI, USA) equipped with a liquid nitrogen quadruple jet thermal modulator and a secondary oven. The first dimension column was a low-polarity crossbond^®^ silarylene phase exhibiting similar selectivity to 5% phenyl/95% dimethyl polysiloxane phases (Rxi^®^-5Sil MS; 30 m × 0.25 mm i.d. × 0.25 μm film thickness; Restek Corp., Bellefonte, PA, USA) connected by means of a deactivated universal Press-Tight^®^ connector (Restek Corp.) to the second dimension. The ^2^D GC column consisted in a medium polarity 50% phenyl polysilphenylene-siloxane phase (BPX50; 1.2 m × 0.10 mm i.d. × 0.10 μm film thickness; SGE International, Victoria, Australia). The ^2^D column was installed in the separate oven located inside the main GC oven, providing more flexible temperature control. The carrier gas was helium at a constant flow rate of 1 mL min^−1^ and the injector split ratio of the split/splitless injector was set to 1:20. The main oven temperature was ramped from 45 °C to 85 °C at 20 °C min^−1^ and then to 285 °C at 5 °C min^−1^, with a final isothermal period of 10 min at 285 °C. The secondary oven was programmed with a 5 °C offset above the primary oven. The modulation period was 4 s and a modulator temperature offset of 15 °C above the main oven was applied. The hot pulse duration was set at 600 ms. Mass spectra were acquired in the range *m/z* 30–400 at an acquisition rate of 100 spectra s^−1^. The ion source temperature was set at 230 °C and the transfer line temperature was set at 250 °C. The detector voltage was 1,500 V and the ionization electron energy (EI source) was set at 70 eV. Samples were acquired using LECO ChromaTOF^®^ software version 4.32. A solvent acquisition delay of 6 min was used to protect the MS analyzer from excessive solvent exposure.

### 3.3. Data Processing

Data were processed using LECO ChromaTOF^®^ software version 4.33. Automatic peak finding with mass deconvolution were used to create a raw peak table, based on minimum signal to noise ratio of 100 and library matches as requirements for a peak to be included in peak tables. The signal to noise ratio was based on the so-called “unique mass”, the most specific mass extracted for an analyte after deconvolution of the MS signal. Further classification processes were applied to remove the chromatographic noise (column bleed) and potential peak tailing issues. Library searching was carried out using NIST/EPA/NIH Mass Spectral Library (NIST 11) and Wiley Registry of Mass Spectral Data (9th Edition). Library similarity factors were reported on a scale of 1,000 unit, the higher the match factor, the better the match, for both forward and reverse searches. Linear retention indices in the first dimension (*I*^T^) were calculated within the ChromaTOF^®^ software using retention times observed for alkane mixture C_8_–C_20_ (Fluka, Belgium) analyzed under the same chromatographic conditions as for samples. The relative deviation of all *I*^T^ obtained in the experiments is lower than 3% according to the 5% phenyl methyl column indices previously reported for these compounds. The first and the second dimension retention times, *I*^T^ and mass spectrometry data were used for compound identifications. Relative abundances (% values) calculations of the compounds were based on the ratio between the peak area of each compound and the sum of areas of all selected compounds.

## 4. Conclusions

Although our previous report on the GC-MS analysis of distillate fractions collected at different times of distillation of mature ylang-ylang flowers freshly harvested permitted the update of the chemical composition of ylang-ylang essential oils, the present study demonstrates the possibility to extract more information when GC×GC-TOFMS is used to separate the hundreds of components of the oil. The gain in information on additional individual compounds has a potential interest in enhancing the statistical treatment of the data by highlighting subtle differences between samples, further improving the chemical distinction between extracts of essential oils originating from different geographical locations. On a more fundamental basis, enlarging the list of specific compounds and gaining a better description of chemical polymorphisms could ultimately contribute to detect adulterations or to study the role of the different components of “terroir” effect.
